# Psychological problems, biomedical models, and stigma: A commentary on Lahey et al. (2022)

**DOI:** 10.1002/jcv2.12119

**Published:** 2022-11-19

**Authors:** Stephen P. Hinshaw

**Affiliations:** ^1^ University of California Berkeley California USA; ^2^ University of California, San Francisco San Francisco California USA

**Keywords:** mental illness, psychological problems, stigma

**Key points**

- The article of Lahey et al. (2022) made a convincing case that quantitative/dimensional, as opposed to categorical/binary, designations of mental health problems are superior in terms of reliability and validity.
- The current commentary offers questions to other contentions–namely, that removing all reference to biomedical underpinnings will automatically reduce stigma.

In a provocative article, Lahey et al. (2022) advocate that, on the basis of substantial research evidence plus the potential to reduce stigma, the field should eliminate psychiatric diagnostic categories in favor of a dimensionalized approach to what should be termed “psychological problems” (as opposed to labels of mental disorders or psychopathology). More boldly, their argument includes a case for eschewing any underlying medical model of such psychological problems.

In this commentary I begin by lauding their key arguments with respect to quantitative and dimensional perspectives. I then proceed to question the view that medical models are inherently stigmatizing, so long as integrative (rather than reductionistic) perspectives are at the forefront. Indeed, even in medicine there is no single medical model (think, for example, of infectious diseases vs. chronic health conditions linked with heritable risk compounded by toxic exposures). That is, a purely behavioral and psychological account is not only overly simplistic but also potentially stigmatizing. Indeed, unintended consequences may emerge from calling all that is typically subsumed under mental disturbance as problems in living or psychological issues. I highlight the existing evidence on consequences of eschewing biomedical conceptions of risk and etiology with respect to both public attitudes and self‐perceptions of those experiencing serious life impairments related to psychological and behavioral functioning.

## KUDOS

Bravo to the authors for an articulate and convincing review of the clear superiority of dimensional accounts regarding the underlying problems (or in a medical view, symptoms) of psychological disturbance. Space limitations preclude a substantive summary, but I highlight the authoritative sources cited by Lahey et al. (2022), which emphasize the non‐appearance of true categories/taxa in systematic literature reviews—along with the polygenic (i.e., multiple genes of small effect) nature of the genetic underpinnings of most neurodevelopmental and mental conditions. Data from the authors' own labs are also highly persuasive, including findings related to the greater reliability and validity of quantitative versus categorical perspectives. Even more, taxonic conceptions (e.g., major depressive disorder; post‐traumatic stress disorder; attention deficit hyperactivity disorder) directly imply that all individuals receiving such diagnoses are fundamentally similar, which is simply untrue on a number of grounds. Heterogeneity rules.

So, a reader might be asking, why not drop any and all categorical taxonomies and adopt a fully dimensionalized perspective, especially one that avoids any disease‐oriented accounts?

## CAVEATS

On the basis of voluminous evidence, it's apparent that categorical diagnoses are arbitrary, relatively unreliable, and insufficiently valid. However, in medicine (e.g., high blood pressure/hypertension), in ascertainment of psychosocial risk (e.g., poverty levels; adverse childhood experiences [ACEs]), or in clinical psychology and psychiatry (e.g., number of relevant problems or symptoms), binary designations are often needed as to who surpasses a given cutoff. Such thresholds are increasingly determined through available data—for example, plotting systolic and diastolic blood pressure readings against eventual stroke risk; plotting poverty levels or ACE scores against impairments in later life; or plotting numbers of attention deficit hyperactivity disorder (ADHD) symptoms against risk for school failure or peer rejection. Note that a host of social and cultural factors may be related to (a) differential cut scores for differing subgroups and (b) the make‐up of the eventual criterion scores linked to impairment. For a recent, extremely lucid article on the promise and perils of precision mental health, see Szatmari and Susser (2022).

Healthcare systems are based on yes/no, diseased/healthy conceptions of illness. In the article's last section, while still advocating for a dimensionalized view, Lahey et al. (2022) explicitly address the categorical question of “who needs treatment” as a driving force for the status quo, stating that challenges of changing the health care system to a fully dimensional approach would be “enormous.” Even more, they assert that *anyone* asking for help with psychological problems should receive reimbursable intervention, perhaps saving societal money in the long run given the pernicious consequences of serious psychological issues.

I believe that the term “enormous” here may be an understatement. Even more, who's to say, absent thresholds, whether more educated and affluent individuals and parents might not demand services for relatively mild psychological issues, while less‐enfranchised members of the population, with far greater needs, would in the process be denied needed care?

Crucially, I believe that the target article's insistence on terming the issues at hand as “psychological problems”—eschewing any kind of underlying medical model with the goal of reducing stigma—may be misguided in key respects. As someone deeply concerned with the reduction of stigma and discrimination, the enhancement of access to services, and the promotion of humanization (Hinshaw, 2017; Martinez & Hinshaw, 2016), I provide some additional thoughts.

First, as Lahey et al. (2022) explicitly admit, there are undoubted biogenetic influences on psychological functioning. For example, heritability estimates for diagnoses and underlying dimensions related to autism (lack of social interest/skill), ADHD (inattention, impulsivity), and bipolar disorder (severe emotion dysregulation) are extraordinarily high, in the range of 0.8–0.9. The heritable risk for many other conditions/dimensions is of at least medium strength. To contend that related functioning is simply “psychological” (which might connote “intentional”) cannot explain the full set of risks and may add to unnecessary self‐blame. I hasten to highlight that, even with substantial heritability figures for many if not most types of mental dysfunction, psychosocial (familial and contextual) forces are still essential for individual development and subsequent life outcomes. In fact, because of gene‐environment interplay, we cannot divorce heritable risk from environmental factors, personal characteristics such as treatment motivation, and systems‐level forces (e.g., treatment access; discrimination).

Second, regarding public stigma, it might be thought that a biogenetic (i.e., a “disease like any other”) portrayal of psychological problems would, through attribution theory, promote benign attitudes because the relevant problems would be seen as beyond the individual's control. In a lucid summary of relevant correlational and experimental research, Haslam and Kvaale (2015) instead propose a “mixed blessings” model, whereby ascriptions of disturbed behavior to exclusively biogenetic causal forces do, in fact, promote reduced public blame but at the same time predict pessimism (“the condition is immutable”), perceptions of danger (“a genetic monster”), and reduced desire for social contact (“keep clear”). Yet do we wish to revert to former notions of psychological problems as products of animal or evil spirits, weak moral fiber, or incompetent parenting (see the “refrigerator parent” theory of autism or the hypothesis that schizophrenia emerges from a “schizophrenogenic mother”)? Of course not. For fascinating and complex empirical data on the pros and cons of mental illness labels and ascriptions, see Rusch et al. (2012), which I cannot review in detail here.

What's needed is a fully integrated and integrative perspective, not a *psychological problems* versus *mental illness* dichotomization. Coronary artery disease (CAD)—which involves the building of plaques inside crucial arteries supplying blood to the heart—is a key trigger for myocardial infarctions (heart attacks). The heritability of CAD is almost exactly 0.5, meaning that individual differences in CAD risk are explained about half by genetic differences and half by environmental/contextual differences across people. But do we perceive those with CAD as less than human, fundamentally flawed? Typically not. Because, however, the brain (with its intricate connections to the rest of the body) is the “seat” of personality and emotion, views of a fundamental lack of humanity still cling to atypical behavior, views that are potentially exacerbated by an exclusive lens on flawed genes.

At the root here is *essentialism*: the belief that a person with a psychological, mental, or physical illness is qualitatively and essentially distinct from other humans (Haslam & Kvaale, 2015). Consider here the longstanding depictions of people with a range of physical disabilities. During much of the 20th Century, such perspectives dominated with respect to cancer, deemed to be a psychosomatic illness triggered by the individual's loss of will to live. Today, with far more basic pathophysiologic knowledge, we understand cancer as a condition to be fought and treated via evidence‐based interventions along with a host of individual family and social supports. Realizing the biological issues underlying much severe psychological dysfunction, while simultaneously considering the individual's fundamental humanity, lies at the core of anti‐stigma efforts. We must fundamentally challenge the assumption that people who struggle with identity, self‐concept, mood regulation, and emotional control (indeed, the clear majority of the population at one or more times of their lives, as noted by Lahey et al., 2022) are inherently and irrevocably flawed, even subhuman.

A recent systematic review and meta‐analysis (Peter et al., 2021) reveals that when individuals view mental disorders (especially conditions like depression) as lying on a continuum with normative affect and behavior, their levels of stigmatization toward such conditions decrease. Yet the same might not apply across the board, especially when severe irrationality, including psychosis, is involved. In fact, in a key finding, Pescosolido et al. (2021) revealed a recent, unprecedented U.S. population‐level change in stigma toward depression, with relatively strong reductions in desire for social distance. Yet within the same data, public attitudes/desired social distance regarding schizophrenia and substance use/addiction had not budged or actually worsened across the past two decades. Progress is not universal.

Judging what's behaviorally normative in a given culture is surely more subjective than ascertaining what's an unhealthy level of coronary artery plaque. In fact, we can never divorce judgments of behavior from social and cultural norms. I urge a non‐reductionist and integrative appeal to consider biological *and* cultural, individual *and* contextual views of behavioral and emotional problems, which require serious investment of resources including true parity and enforcement of anti‐discrimination policies. Treatments need to promote strengths as well as remediate areas of deficit, by promoting social connectedness and making evidence‐based accommodations in schools and in the workplace. We can no longer afford to discount and waste human potential in the face of emotional and behavioral challenges by thinking reductionistically. The future of our interdependent species is at stake.

## CONCLUSION

If our ultimate goal is to help individuals, families, and cultures thrive, to drive our economies, and to promote a more harmonious world, we must address psychological distress and pain with the realization that humans lie on multiple spectra of core dimensions of functioning and well‐being. Most forms of psychological disturbance/mental dysfunction are common, waxing and waning over months and years, with recovery a true possibility. Research continues to reveal, especially for the most severe and impairing forms of such conditions, clear biomedical underpinnings that always operate and transact with family, social, and cultural systems. The aims of recognition (and, when needed, use of the most evidence‐based thresholds), provision of evidence‐based treatment, promotion of strengths despite areas of impairment, and insistence on humanization will be not be well served by an “either/or” in terms of biological risk versus psychological functioning and cultural contexts. Rather, to promote humanization, inclusion, and belonging, our perspectives must be “both/and.”

## AUTHOR CONTRIBUTIONS

**Stephen P. Hinshaw**: Conceptualization; Writing – original draft; Writing – review & editing.

## CONFLICT OF INTEREST

The author has declared that he has no competing or potential conflicts of interest.

## Data Availability

No data analyzed in this narrative Commentary.
